# POSTAR3: an updated platform for exploring post-transcriptional regulation coordinated by RNA-binding proteins

**DOI:** 10.1093/nar/gkab702

**Published:** 2021-08-17

**Authors:** Weihao Zhao, Shang Zhang, Yumin Zhu, Xiaochen Xi, Pengfei Bao, Ziyuan Ma, Thomas H Kapral, Shuyuan Chen, Bojan Zagrovic, Yucheng T Yang, Zhi John Lu

**Affiliations:** MOE Key Laboratory of Bioinformatics, Center for Synthetic and Systems Biology, School of Life Sciences, Tsinghua University, Beijing 100084, China; MOE Key Laboratory of Bioinformatics, Center for Synthetic and Systems Biology, School of Life Sciences, Tsinghua University, Beijing 100084, China; MOE Key Laboratory of Bioinformatics, Center for Synthetic and Systems Biology, School of Life Sciences, Tsinghua University, Beijing 100084, China; Department of Maternal, Child and Adolescent Health, School of Public Health, Anhui Medical University, MOE Key Laboratory of Population Health Across Life Cycle, NHC Key Laboratory of Study on Abnormal Gametes and Reproductive Tract, Anhui Provincial Key Laboratory of Population Health and Aristogenics, No 81 Meishan Road, Hefei 230032, Anhui, China; MOE Key Laboratory of Bioinformatics, Center for Synthetic and Systems Biology, School of Life Sciences, Tsinghua University, Beijing 100084, China; MOE Key Laboratory of Bioinformatics, Center for Synthetic and Systems Biology, School of Life Sciences, Tsinghua University, Beijing 100084, China; MOE Key Laboratory of Bioinformatics, Center for Synthetic and Systems Biology, School of Life Sciences, Tsinghua University, Beijing 100084, China; School of Pharmaceutical Sciences, Tsinghua University, Beijing 100084, China; Department of Structural and Computational Biology, Max Perutz Labs, University of Vienna, Campus Vienna Biocenter 5, A-1030 Vienna, Austria; MOE Key Laboratory of Bioinformatics, Center for Synthetic and Systems Biology, School of Life Sciences, Tsinghua University, Beijing 100084, China; Faculty of Science, University of Melbourne, Melbourne, Victoria 3010, Australia; Department of Structural and Computational Biology, Max Perutz Labs, University of Vienna, Campus Vienna Biocenter 5, A-1030 Vienna, Austria; Institute of Science and Technology for Brain-Inspired Intelligence, Fudan University, Shanghai 200433, China; MOE Key Laboratory of Computational Neuroscience and Brain-Inspired Intelligence, Fudan University, Shanghai 200433, China; MOE Frontiers Center for Brain Science, Fudan University, Shanghai 200433, China; MOE Key Laboratory of Bioinformatics, Center for Synthetic and Systems Biology, School of Life Sciences, Tsinghua University, Beijing 100084, China

## Abstract

RNA-binding proteins (RBPs) play key roles in post-transcriptional regulation. Accurate identification of RBP binding sites in multiple cell lines and tissue types from diverse species is a fundamental endeavor towards understanding the regulatory mechanisms of RBPs under both physiological and pathological conditions. Our POSTAR annotation processes make use of publicly available large-scale CLIP-seq datasets and external functional genomic annotations to generate a comprehensive map of RBP binding sites and their association with other regulatory events as well as functional variants. Here, we present POSTAR3, an updated database with improvements in data collection, annotation infrastructure, and analysis that support the annotation of post-transcriptional regulation in multiple species including: we made a comprehensive update on the CLIP-seq and Ribo-seq datasets which cover more biological conditions, technologies, and species; we added RNA secondary structure profiling for RBP binding sites; we provided miRNA-mediated degradation events validated by degradome-seq; we included RBP binding sites at circRNA junction regions; we expanded the annotation of RBP binding sites, particularly using updated genomic variants and mutations associated with diseases. POSTAR3 is freely available at http://postar.ncrnalab.org.

## INTRODUCTION

RNA-binding proteins (RBPs) are essential regulators of RNA function in various biological processes (1,2) and are especially critical in post-transcriptional regulation (3–5). In recent years, several high-throughput sequencing technologies based on crosslinking and immunoprecipitation (CLIP) have been developed to detect genome-wide RBP binding sites (6,7). Moreover, we are able to investigate RNA secondary structure *in vivo* using secondary structure profiling (structure-seq) (8–10), and degradation of cellular RNAs caused by bound miRNAs using degradome sequencing (degradome-seq) (11–13). Together with these high-throughput sequencing technologies, RBP binding could be associated with RNA secondary structure and other types of post-transcriptional regulation events, which would be helpful to understand the post-transcriptional regulation networks that are coordinated by RBPs. Previous studies have revealed the relationship between RBP binding and RNA secondary structure (14,15), as well as miRNA-mediated degradation (16). Furthermore, other studies have shown that RBP played an important role in circRNA formation and function (17,18). A platform summarizing RBP binding sites recovered by CLIP-seq and other post-transcriptional regulation events would definitely be helpful for the study in the field.

We have developed a series of CLIPdb/POSTAR databases that focus on the functional annotations of RBP binding sites, as well as their association to other types of post-transcriptional regulation events (19–21). As both the types and volume of these high-throughput dataset have dramatically increased in recent years, it is imperative to update the database to a new version, curating more comprehensive information for RBP binding and post-transcriptional regulation. Here, we present POSTAR3, an update to our existing database of RBP binding records and RNA post-transcriptional regulation (19–21). POSTAR3 curated 339 new CLIP-seq datasets, which spanned nine CLIP-seq technologies from human and other six model species, as well as 300 Ribo-seq datasets covering ∼100 tissue types, cell lines, developmental stages, and experimental conditions from six species, 82 secondary structure profiling datasets, and 83 degradome-seq datasets paired with small RNA sequencing (sRNA-seq) data. We also included RBP binding sites on circRNA junction regions. We associated the RBP binding sites identified from CLIP-seq datasets with other levels of information, including RNA post-transcriptional regulation, genomic variants, disease-associated mutations, secondary structure profile and model, and miRNA-mediated decay from various sources. We also re-designed and modified our database interface to provide an informative display of different types of data and a valuable platform to explore their relationship. We expect that POSTAR3 would be a valuable resource and platform for researchers to investigate post-transcriptional regulation, RNA secondary structure dynamics, miRNA-mediated decay, and their relationship with RBP binding.

## DATA COLLECTION UPDATES AND DATA PROCESSING

### Updates on the CLIP-seq dataset collection

**Figure 1.**
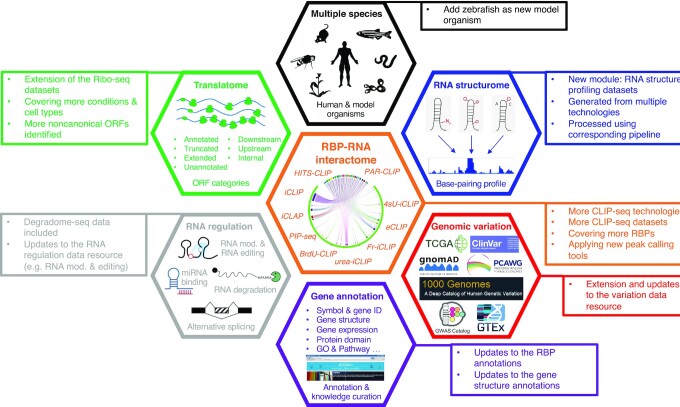
Overview of POSTAR3 database content. Our database is concentrated in RBP-RNA interaction network and reveals information related to RBP binding through CLIP-seq. Other types of post-transcriptional regulation events (RNA modification and editing, genomic variants, disease-associated mutations, secondary structure profile, miRNA-mediated decay, etc.) and translational dynamics from Ribo-seq is associated with RBP binding in order to give users novel insights to the relationship between these events.

**Figure 2.**
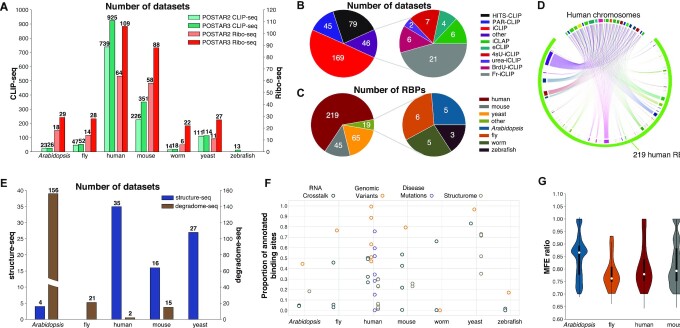
Statistics of data curated in POSTAR3 database. (**A**) Number of CLIP-seq and Ribo-seq datasets in seven species, compared with our previous version POSTAR2. (**B**) Number of newly curated CLIP-seq datasets using different technologies. (**C**) Number of curated RBPs in seven species. (**D**) RBP-RNA interactome network of human in POSTAR3. Arcs on the top represents chromosomes in human, and bottom ones represents RBPs. (**E**) Number of structure-seq and degradome-seq datasets curated in POSTAR3. (**F**) Annotation status of RBP binding sites in different modules. Each dot indicates a specific set of data. (**G**) MFE ratio distribution in all degradome duplex across 4 species.

To expand the spectrum of RBP binding events in our database, we manually collected 339 new publicly available CLIP-seq datasets that used CLIP-seq technologies from Gene Expression Omnibus (GEO) (22), Sequence Read Archive (SRA) (23), ArrayExpress (24), and DDBJ Sequence Read Archive (DRA) (25) (Supplementary Table S1 and Supplementary Table S2). We also updated ENCODE eCLIP to the latest release (26,27), which contains 225 eCLIP datasets from 150 RBPs (Supplementary Table S3). By combining the binding sites from our new datasets with our previous records (21), POSTAR3 contains 1499 CLIP-seq datasets from 348 RBPs in total (Figure 1 and Supplementary Table S1), which is a significant improvement in terms of the number of CLIP-seq datasets as well as the RBPs covered (Figure 2A). In summary, comparing to the four CLIP-seq technologies in POSTAR2, POSTAR3 has covered 10 various CLIP-seq technologies (i.e. HITS-CLIP, PAR-CLIP, iCLIP, eCLIP, iCLAP, urea-iCLIP, 4sU-iCLIP, BrdU-CLIP, Fr-iCLIP and PIP-seq). In total, it includes 348 RBPs from seven species (i.e. human, mouse, zebrafish, fly, worm, *Arabidopsis* and yeast) (Figure 2B and C). To our knowledge, POSTAR3 provides the largest collection of RBP binding sites from diverse CLIP-seq technologies and multiple species.

### Identification of RBP binding sites from CLIP-seq datasets

For each newly collected CLIP-seq dataset, we followed the same analysis procedure as we developed in POSTAR2 (21) with some modifications. To improve the read mapping quality, we removed unique molecular identifier (UMI) in the raw sequencing file using FASTX-Toolkit (http://hannonlab.cshl.edu/fastx_toolkit). The actual number of nucleotides that needed to be removed was determined according to the description in the original publications. We also updated the technology-specific peak callers: we used CLIPper (28) (human)/CTK (29) (other species) for HITS-CLIP related technology (HITS-CLIP, BrdU-CLIP), MiClip (30) for PAR-CLIP, and PureCLIP (31) for iCLIP related technology (iCLIP, eCLIP, iCLAP, urea-iCLIP, 4sU-iCLIP, Fr-iCLIP) with default parameters (Supplementary Table S4). For ENCODE eCLIP datasets, we obtained the binding sites from the ENCODE data portal (https://www.encodeproject.org/, May 2020). We also downloaded human RBP binding sites on circRNA junction regions from several recent studies (32,33) and converted the region coordinates to hg38 using liftOver (34). The binding records curated in our database enabled us to construct an RBP-RNA interactome network (Figure 2D).

### Adding structure-seq datasets

In POSTAR3, we added a novel ‘Structurome’ module, where we collected 66 structure-seq datasets (Supplementary Table S5) from GEO (22) and SRA (23) database (Figure 2E), and processed the data as in the original publications. We also collected six processed icSHAPE datasets (Supplementary Table S6) from ENCODE (35). After we obtained the base-pairing information from these datasets, we tried to predict the secondary structure model around RBP binding sites. We extended the RBP binding sites to 150nt flanking the midpoint, and extracted the genomic sequences from the genomes of their respective species as well as the matched structural profiles. Notably, we did this calculation only for the binding sites on long RNAs. We then predicted the RNA secondary structure using Fold from RNAstructure (36) and RNAfold from ViennaRNA (37) with default parameters, in which the structural profile was used as soft constraint. Together with other annotations, POSTAR3 provides users with enough resources to investigate the relationship between RBP binding and other types of post-transcriptional regulatory events (Figure 2F).

### Updates of Ribo-seq datasets

We have collected 129 new Ribo-seq datasets (Supplementary Table S7), as well as their matched RNA-seq datasets (Supplementary Table S8) from GEO (22) and SRA (23) database (Figure 2A). We followed the processing procedure from our previous paper (21), with modifications as follows. We used RiboCode (38) to process Ribo-seq mapped reads and identify all types of putative open reading frames (ORFs). We then used Ribotaper (39), ORFscore (40) and RibORF (41) to identify and evaluate translated ORFs in the newly collected datasets. The translation efficiency of the ORF was defined as the RPKM ratio of Ribo-seq to the paired RNA-seq. We obtained the RPKM values of the ORFs based on the raw read density from Ribo-seq datasets, as well as the processed read density from RiboCode (38).

### Adding Degradome-seq datasets

In POSTAR3, we also added a Degradome module, where we collected 83 degradome-seq datasets (Supplementary Table S9) and 111 matched small RNA-seq (sRNA-seq) datasets (Supplementary Table S10) from public resource (Figure 2E). To avoid false discovery and unnecessary bias, we excluded datasets without raw fastq files or matched sRNA-seq datasets. Briefly, we removed the adapter sequence using Cutadapt (42) and filtered low quality samples based on the trimming results using FastQC (https://www.bioinformatics.babraham.ac.uk/projects/fastqc/). The cleaned fastq files of sRNA-seq datasets were subsequently aligned to annotated miRNA sequences using bowtie2 (43) with the following parameters: -p 12 -n 0 -m 5 –best –strata. We then identified miRNA-mediated degradation events with fastq files converted from sRNA bam files and cleaned degradome-seq fastq files using PAREsnip2 (44) with the stringent mode, Carrinton rule, and the corresponding transcriptome annotations. In addition, we found that the Minimum Free Energy (MFE) ratio (actual binding MFE versus theoretical MFE) of the duplex regions are relatively high in the four species (i.e. human, mouse, fly and *Arabidopsis*) (Figure 2G).

### Updates on the annotations of RBP and RBP binding sites

Other than the RBP binding sites itself, we also made significant efforts to update the annotation of RBPs and RBP binding sites. We added annotation information for newly-added RBP and binding sites from zebrafish. We also retrieved information on circRNA from circBase (45) and miRNA from miRbase (46) to annotate respective RNAs. We included overexpression information of the RBP in respective CLIP-seq experiments. We added ∼78 million SNV from 1000 genomes (47), ∼679 million SNV from gnomAD (48), ∼40 million eQTLs, and ∼16 million sQTLs from GTEx (49,50) to annotate RBP binding sites with genomic variants, as well as ∼906k CCLE (51) variants, ∼406k denovo-db (52) variants and ∼7k HmtDB (53) variants as disease-associated mutations. Detailed annotation process for RBP and RBP binding sites is described in Supplemental Methods.

## DATABASE FEATURES AND APPLICATIONS

### Database and website architecture

All data in POSTAR3 were processed and stored in a MySQL Database (version 5.6.50). We implemented the client-side user interface by the HTML5 and JavaScript libraries, including jQuery (http://jquery.com) and Bootstrap (http://getbootstrap.com), and the server-side using PHP scripts (version 5.6) and JavaScript. Plots of query results in POSTAR3 were generated by plotly.js library (https://plot.ly) and Highcharts (https://www.highcharts.com). Tables of query results were produced by the DataTables JavaScript library (https://www.datatables.net) that allows users to search and sort results. We generated RNA secondary structure visualization by forna (54). We used UCSC Genome Browser (34) to visualize genome in our website. We have tested the web page in several popular browsers including Google Chrome, Safari, Microsoft Edge and Firefox. Users could get access to the website link either on a computer or mobile device.

### Overview of the web interface

In POSTAR3, we have updated the website design, which provides a user-friendly web interface for searching, browsing, and downloading data from seven species and eight modules. Here, we briefly describe the implementation of each module.

The ‘CLIPdb’ module provides the annotation of RBPs with their binding sites identified from CLIP-seq datasets. In POSTAR3, we have updated the annotation for the query RBP such as RNA recognition domains, RBP ontology, sequence motifs, and structural preferences in this module. We also provided the overexpression status of the RBP in the original experiment when searching for RBP binding sites. The ‘RBP Binding Sites’ module displays all the RBP binding sites identified with different CLIP-seq technologies and peak calling methods when searching the target gene. The table and network view present the interaction between RBPs and target genes. We also collected genomic location, associated diseases, and expression patterns across different cell lines, tissue types, developmental stages, or conditions for annotation of the target gene. Notably, we generate an overview of the high-occupancy target regions by defining the ‘RBP binding hotspots’ according to the number of RNA binding sites of each 20nt bin on the RNA’s precursor. The ‘RNA Crosstalk’ module provides the interactions between RBP binding sites and other post-transcriptional regulation events, including miRNA targets, RNA modification, and RNA editing. The ‘Genomic Variants’ module and the ‘Disease Mutations’ module integrate SNVs and disease-associated mutations with RBP binding sites to provide insight into the causal variants and the underlying regulatory mechanisms of human diseases. The ‘Translatome’ module characterizes the translation landscape of RNAs with one summary frame and three tables for seven categories of ORFs, respectively. For each data table in POSTAR3, we provide ‘Export data to CSV file’ option for users to download the results of the whole table. Moreover, to provide users with a convenient view of different modules in our database, we have also constructed a ‘POSTAR3 Central’ page. At the bottom of each RNA-centric module, there is a link to this ‘POSTAR3 Central’ page. Users could click the link to enter this page and transfer to other modules by clicking the respective link.

We would like to highlight another two new modules that are included in POSTAR3. The new ‘Structurome’ module is constructed for characterizing the secondary structure landscape of RNAs. Users can choose a species (e.g. human, mouse, zebrafish, fly, worm, *Arabidopsis* or yeast) and input the desired gene name. POSTAR3 then returns a genome browser, a network and a table: the genome browser contains regions for predicted secondary structure and RBP binding sites corresponding to the table; the network represents interacting RBPs with the queried RNA; the table presents structure information of RBP binding sites for the searched gene. Reactivity score and RNA secondary structure are plotted at each row in the table. The ‘Degradome’ module provides binding information between miRNA and other types of RNA which leads to the degradation of the other RNA validated by degradome-seq data. Users can obtain detailed information about every validated sRNA-fragment pair by selecting a species and input a target RNA name or small RNA name.

### Example applications

POSTAR3 provides users with a friendly and informative platform for exploring the relationship between RBP binding and various types of post-transcriptional regulation events, genomic variants, and translational dynamics. Here, we present two example applications using our database, particularly the two new modules, to demonstrate how to decipher potential regulatory mechanisms related to human disease and response to external stimuli in plants.

**Figure 3.**
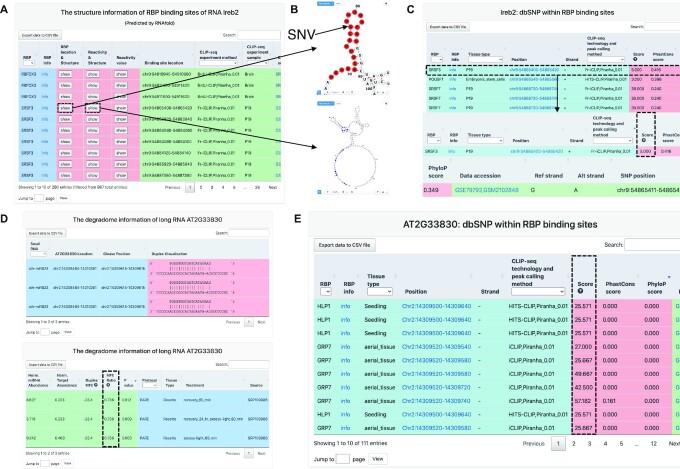
Example applications of POSTAR3: studying Ireb2 in mouse and AT2G33830 in *Arabidopsis*. (**A**) Search of mouse Ireb2 gene in ‘Structurome’. In the ‘Structurome’ module, users could observe the secondary structure model predicted by algorithms enhanced by secondary structure profiling data. (**B**) They could also click the ‘RBP location & Structure’ or ‘Reactivity & structure’ button to visualize secondary structure using forna, along with other layers of information. (**C**) Search of mouse Ireb2 gene in ‘Disease Mutations’ module. ‘Disease Mutations’ module provides users with information of disease-associated mutations associated with RBP binding in human. Notice that the score for this binding site was relatively high. (**D**) Search of *Arabidopsis* AT2G33830 gene in ‘Degradome’ module. Search in ‘Degradome’ module returns a table containing knowledge of miRNA–mRNA binding and degradation peaks, with statistical scores indicating the reliability of the degradation pair. (**E**) Search of *Arabidopsis* AT2G33830 gene in ‘Genomic Variants’ module. ‘Genomic Variants’ module gives us information on genomic variants resided within the RBP binding sites.

In the first example, Ireb2 (also known as Irp2) encodes an essential iron responsive element binding protein in mouse, and its homologous gene has been reported to be related to iron homeostasis in human cells (55). Further studies in mice revealed that Ireb2 could regulate insulin production by influencing iron levels and triggering downstream biochemical reactions (56). However, little effort has been made to demonstrate the relationship between RBP binding and RNA post-transcriptional regulation, especially the secondary structural change during response to iron and production of insulin. When we queried ‘Ireb2’ in ‘Structurome’ module in our database, the website returned a genome browser showing the position of RBP binding sites, a network view of interacting RBP of this RNA, and a table displaying all the binding sites and its secondary structure model enhanced by structure profiling data (Figure 3A). In one of the SRSF3 binding sites on Ireb2, we could observe that the binding site was placed at a stem-loop structure (Figure 3B). At the same time, if we query ‘Ireb2’ in ‘Genomic Variants’ module, we could retrieve genomic variation information coordinated with RBP binding sites, including one SNV event from dbSNP in this binding site, while the score for the RBP binding site was relatively high (Figure 3C). This variant caused a G changing to an A, thus affecting the secondary structure of this local binding site. These results suggest that this variation could have putative association with the secondary structure change of Ireb2 mRNA, thus influencing the binding of SRSF3, and further affect insulin production and development of diabetes in mouse and human.

Another example is AT2G33830 (also known as DRM2) in *Arabidopsis*. Recent studies have revealed that the expression of AT2G33830 could be related to plants’ response to stress and external stimuli, including response to light (57). However, the mechanism of controlled AT2G33830 expression has not been fully understood. When we searched ‘AT2G33830’ in the new ‘Degradome’ module, the database returned a table containing peaks of miRNA binding and degradation in degradome-seq data (Figure 3D). All these peaks were identified from a study that investigate the response to excessive light in plants (58), with a relatively high MFE ratio, suggesting stable degradation pairs were formed between the miRNA and the target RNA. Meanwhile, if we search AT2G33830 in the ‘Genomic Variants’ module, one SNV was found in the base pairing region bound by miRNA, where multiple RBP binding sites with high binding score resided around this region (Figure 3E). Taking all these results together, we could propose a possible mechanism of light response in *Arabidopsis* that the expression of AT2G33830 can be regulated by miRNA binding and degradation, and also affected by SNPs and RBP binding in this local region.

## DISCUSSION AND FUTURE DIRECTIONS

We systematically updated our database to the new version, POSTAR3, to enable users to make discoveries and decipher regulatory mechanisms underlying post-transcriptional regulation events related to RBPs. POSTAR3 records ∼50 million RBP binding sites from seven species (human, mouse, zebrafish, fly, worm, *Arabidopsis*, and yeast) and diverse CLIP-seq technologies (HITS-CLIP, PAR-CLIP, iCLIP, PIP-seq, eCLIP, iCLAP, urea-iCLIP, 4sU-iCLIP, BrdU-CLIP, Fr-iCLIP). To our knowledge, POSTAR3 provides the largest collection of RBP binding sites that are identified from CLIP-seq datasets. We annotated the binding sites by incorporating other high-throughput sequencing data, including Ribo-seq, RNA secondary structure profiling, and degradome-seq, as well as other types of post-transcriptional regulation events and genomic variants, shedding light on the relationship between RBP binding and regulatory mechanism at the post-transcriptional and translational level.

Compared with our previous release of POSTAR2, POSTAR3 has made the following updates and improvements: (i) POSTAR3 provides more RBP binding sites that are identified from CLIP-seq datasets and ORFs recovered from Ribo-seq datasets, covering more species and experimental technologies; (ii) POSTAR3 contains two new modules: ‘Structurome’ and ‘Degradome’, which provide secondary structure profiling data and model of RBP binding sites, and sRNA-fragment binding records leading to degradation of other RNAs validated by degradome-seq; (iii) POSTAR3 curates RBP binding sites on circRNA junction regions that were recovered from CLIP-seq datasets; (iv) POSTAR3 added annotation information for RBPs, especially the overexpression status information in each CLIP-seq experiment; (v) POSTAR3 updates the annotation for RBP binding sites, including post-transcriptional regulation events, genomic variants, and disease-associated mutations; (vi) POSTAR3 re-designed and modified our website to build a user-friendly interface for scientists. Since mobile devices are now used more and more widely, we also invested efforts to ensure a compatible web interface on these devices.

It is noticed that sometimes, there is discrepancy between established motifs and motifs discovered from CLIP-seq data in our database. Nevertheless, in our opinion, this should not be a problem. Most experimental motif discovery methods were *in vitro*, such as SELEX or RNAcompete. However, CLIP-seq experiments were conducted *in vivo*, and it is sometimes difficult to identify motifs from CLIP-seq experiments due to protein cofactor interactions or non-specific background (59). As a result, it is possible that our motif discovery process might not be able to find those established motifs from the in vitro experiments. We followed the process pipeline in previous versions of our database to ensure reliable motif detection.

With the development of novel high-throughput sequencing technologies designed to decode the post-transcriptional regulation and release of high-quality data for all kinds of regulatory events, datasets that cover more species and biological conditions will become available to the public in the near future. We would like to continue to incorporate new high-throughput data and improve website for better navigation and exploration of curated data. We will continue to maintain and update our POSTAR3 database to make sure it remains a useful resource for researchers in this area.

## DATA AVAILABILITY

POSTAR3 is freely available at http://postar.ncrnalab.org (also at http://lulab.life.tsinghua.edu.cn/postar). Data in POSTAR3 can be downloaded and used in accordance with the GNU Public License and the license of their primary data sources.

## Supplementary Material

gkab702_Supplemental_Files
