# Supplementary material for: POSTAR3: an updated platform for exploring post-transcriptional regulation coordinated by RNA-binding proteins
Source: Nucleic Acids Res. 2021 Aug 17;50(D1):D287–94. doi: 10.1093/nar/gkab702 (PMC8728292; doi:10.1093/nar/gkab702)
Supplement: gkab702_Supplemental_Files [file gkab702_supplemental_files.zip › supplementary_materials.pdf]

Supplementary Methods

Annotation of RBP and RBP binding sites

For each RBP in our database, we obtained the protein domains from Pfam (1). GO term annotation of RBP was collected from AmiGO (2,3). We collected RBP category information of human from a recent study (4). To annotate each RBP binding site with its genomic information, we used their respective genome annotation: Gencode V27 (human), Gencode VM7 (mouse), ZFIN danRer11 (zebrafish), Flybase dmel-r6.18 (fly), WormBase ws235 (worm), TAIR10 (*Arabidopsis*), SGD R64-1-1 (yeast) (5-10). In order to comprehensively annotate RBP binding sites located in long non-coding RNAs (lncRNAs), we used Gencode (5) lncRNA annotations for human and mouse, and NONCODE (11) annotations for other species. We collected expression patterns of different species from our previous database (12), and added zebrafish gene expression from Expression Atlas (13). We followed the same definition of "RBP hotspots" in our previous publication (12), and calculated hotspots on each transcript using our new RBP binding site records. In some CLIP-seq experiments, the RBP was overexpressed or induced as a tagged protein, and abnormal expression of the RBP gene would affect the binding preference of the RBP, so we included overexpression information in our database by collecting the information from the original publication. The circRNA annotation was retrieved from circBase (14), including genomic location and expression value of circRNA in different studies. We also annotated miRNA in Degradome module using information in the miRBase (15) and AmiGO (2,3). To annotate RBP with their binding sequence and structural preference, we followed the same procedure in our previous publication (12) to calculate RBP binding sequence and structure motif using new data in POSTAR3. In short, we split the RBP binding sites called by Piranha (16) into independent training and test sets. Then we applied MEME (17) and HOMER (18) on the training sets to identify no more than five sequence motifs for each RBP. Among these five motifs, we selected ones that show the most significant enrichment in the test sets, where they were defined based on p-values from FIMO (19). We visualized the sequence motifs using WebLogo (20). To reveal the structural preference of an RBP, every peak was elongated into 60nt, and we used RNAcontext (21) to detect local structural motifs, including paired (P), hairpin loop (L), bulge/internal/multi-loop (M), and unstructured (U) with the relative preference of each kind of structural motifs. We also used RNApromo (22) to predict

structural elements enriched in the RBP binding area with p-value < 0.05.

Updates of Crosstalk, variation, and disease annotation

To display interactions and regulations of RNAs that were within RBP binding sites, we prepared miRNA binding sites from both computational prediction and experimental validation, RNA editing sites, and RNA modification sites as described in our previous publication (12), and intersected these events with RBP binding sites in our database. As various resource of genomic variants and disease-associated mutations was made public in recent years, we managed to include these data in our database to help researchers investigate problems related to RBP regulation of these variants and their implication on disease. We collected genomic variants from dbSNP (23), 1000 Genomes (24), GTEx eQTLs and sQTLs (25,26), and Genome Aggregation Database (gnomAD) (27). We also curated disease-associated variants and mutations from TCGA PCAWG and PanTCGA (28,29), CCLE (30), ClinVar (31), COSMIC (32), denovo-db (33), GWASdb2 (34), and HmtDB (35). All information was collected, reformatted, and intersected with RBP binding sites to annotate variant information within each RBP binding site.

References

1. Mistry, J., Chuguransky, S., Williams, L., Qureshi, M., Salazar, G.A., Sonnhammer, E.L.L., Tosatto, S.C.E., Paladin, L., Raj, S., Richardson, L.J. *et al.* (2021) Pfam: The protein families database in 2021. *Nucleic Acids Res*, **49**, D412-D419.
2. Gene Ontology, C. (2021) The Gene Ontology resource: enriching a GOld mine. *Nucleic Acids Res*, **49**, D325-D334.
3. Carbon, S., Ireland, A., Mungall, C.J., Shu, S., Marshall, B., Lewis, S., Ami, G.O.H. and Web Presence Working, G. (2009) AmiGO: online access to ontology and annotation data. *Bioinformatics*, **25**, 288-289.
4. Van Nostrand, E.L., Freese, P., Pratt, G.A., Wang, X., Wei, X., Xiao, R., Blue, S.M., Chen, J.Y., Cody, N.A.L., Dominguez, D. *et al.* (2020) A large-scale binding and functional map of human RNA-binding proteins. *Nature*, **583**, 711-719.
5. Harrow, J., Frankish, A., Gonzalez, J.M., Tapanari, E., Diekhans, M., Kokocinski, F., Aken, B.L., Barrell, D., Zadissa, A., Searle, S. *et al.* (2012) GENCODE: the reference human genome annotation for The ENCODE Project. *Genome Res*, **22**, 1760-1774.
6. Ruzicka, L., Howe, D.G., Ramachandran, S., Toro, S., Van Slyke, C.E., Bradford, Y.M., Eagle, A., Fashena, D., Frazer, K., Kalita, P. *et al.* (2019) The Zebrafish Information Network: new support for non-coding genes, richer Gene Ontology annotations and the Alliance of Genome Resources. *Nucleic Acids Res*, **47**, D867-D873.
7. dos Santos, G., Schroeder, A.J., Goodman, J.L., Strelets, V.B., Crosby, M.A., Thurmond, J., Emmert, D.B.,

Gelbart, W.M. and FlyBase, C. (2015) FlyBase: introduction of the Drosophila melanogaster Release 6 reference genome assembly and large-scale migration of genome annotations. *Nucleic Acids Res*, **43**, D690-697.

8. Harris, T.W., Antoshechkin, I., Bieri, T., Blasiar, D., Chan, J., Chen, W.J., De La Cruz, N., Davis, P., Duesbury, M., Fang, R. *et al.* (2010) WormBase: a comprehensive resource for nematode research. *Nucleic Acids Res*, **38**, D463-467.
9. Huala, E., Dickerman, A.W., Garcia-Hernandez, M., Weems, D., Reiser, L., LaFond, F., Hanley, D., Kiphart, D., Zhuang, M., Huang, W. *et al.* (2001) The Arabidopsis Information Resource (TAIR): a comprehensive database and web-based information retrieval, analysis, and visualization system for a model plant. *Nucleic Acids Res*, **29**, 102-105.
10. Cherry, J.M., Hong, E.L., Amundsen, C., Balakrishnan, R., Binkley, G., Chan, E.T., Christie, K.R., Costanzo, M.C., Dwight, S.S., Engel, S.R. *et al.* (2012) Saccharomyces Genome Database: the genomics resource of budding yeast. *Nucleic Acids Res*, **40**, D700-705.
11. Zhao, L., Wang, J., Li, Y., Song, T., Wu, Y., Fang, S., Bu, D., Li, H., Sun, L., Pei, D. *et al.* (2021) NONCODEV6: an updated database dedicated to long non-coding RNA annotation in both animals and plants. *Nucleic Acids Res*, **49**, D165-D171.
12. Zhu, Y., Xu, G., Yang, Y.T., Xu, Z., Chen, X., Shi, B., Xie, D., Lu, Z.J. and Wang, P. (2019) POSTAR2: deciphering the post-transcriptional regulatory logics. *Nucleic Acids Res*, **47**, D203-D211.
13. Papatheodorou, I., Fonseca, N.A., Keays, M., Tang, Y.A., Barrera, E., Bazant, W., Burke, M., Fullgrabe, A., Fuentes, A.M., George, N. *et al.* (2018) Expression Atlas: gene and protein expression across multiple studies and organisms. *Nucleic Acids Res*, **46**, D246-D251.
14. Glazar, P., Papavasileiou, P. and Rajewsky, N. (2014) circBase: a database for circular RNAs. *RNA*, **20**, 1666-1670.
15. Griffiths-Jones, S., Grocock, R.J., van Dongen, S., Bateman, A. and Enright, A.J. (2006) miRBase: microRNA sequences, targets and gene nomenclature. *Nucleic Acids Res*, **34**, D140-144.
16. Uren, P.J., Bahrami-Samani, E., Burns, S.C., Qiao, M., Karginov, F.V., Hodges, E., Hannon, G.J., Sanford, J.R., Penalva, L.O. and Smith, A.D. (2012) Site identification in high-throughput RNA-protein interaction data. *Bioinformatics*, **28**, 3013-3020.
17. Bailey, T.L. and Elkan, C. (1994) Fitting a mixture model by expectation maximization to discover motifs in biopolymers. *Proc Int Conf Intell Syst Mol Biol*, **2**, 28-36.
18. Heinz, S., Benner, C., Spann, N., Bertolino, E., Lin, Y.C., Laslo, P., Cheng, J.X., Murre, C., Singh, H. and Glass, C.K. (2010) Simple combinations of lineage-determining transcription factors prime cis-regulatory elements required for macrophage and B cell identities. *Mol Cell*, **38**, 576-589.
19. Grant, C.E., Bailey, T.L. and Noble, W.S. (2011) FIMO: scanning for occurrences of a given motif. *Bioinformatics*, **27**, 1017-1018.
20. Crooks, G.E., Hon, G., Chandonia, J.M. and Brenner, S.E. (2004) WebLogo: a sequence logo generator. *Genome Res*, **14**, 1188-1190.
21. Kazan, H., Ray, D., Chan, E.T., Hughes, T.R. and Morris, Q. (2010) RNAcontext: a new method for learning the sequence and structure binding preferences of RNA-binding proteins. *PLoS Comput Biol*, **6**, e1000832.
22. Rabani, M., Kertesz, M. and Segal, E. (2008) Computational prediction of RNA structural motifs involved in posttranscriptional regulatory processes. *Proc Natl Acad Sci U S A*, **105**, 14885-14890.
23. Sayers, E.W., Beck, J., Bolton, E.E., Bourexis, D., Brister, J.R., Canese, K., Comeau, D.C., Funk, K., Kim, S., Klimke, W. *et al.* (2021) Database resources of the National Center for Biotechnology Information. *Nucleic Acids Res*, **49**, D10-D17.

24. 1000 Genomes Project Consortium *et al.* (2015) A global reference for human genetic variation. *Nature*, **526**, 68-74.

25. Aguet, F., Brown, A.A., Castel, S.E., Davis, J.R., He, Y., Jo, B., Mohammadi, P., Park, Y., Parsana, P., Segre, A.V. *et al.* (2017) Genetic effects on gene expression across human tissues. *Nature*, **550**, 204-213.

26. Aguet, F., Barbeira, A.N., Bonazzola, R., Brown, A., Castel, S.E., Jo, B., Kasela, S., Kim-Hellmuth, S., Liang, Y.Y., Parsana, P. *et al.* (2020) The GTEx Consortium atlas of genetic regulatory effects across human tissues. *Science*, **369**, 1318-1330.

27. Karczewski, K.J., Francioli, L.C., Tiao, G., Cummings, B.B., Alfoldi, J., Wang, Q.B., Collins, R.L., Laricchia, K.M., Ganna, A., Birnbaum, D.P. *et al.* (2020) The mutational constraint spectrum quantified from variation in 141,456 humans. *Nature*, **581**, 434-443.

28. Alexandrov, L.B., Nik-Zainal, S., Wedge, D.C., Aparicio, S.A.J.R., Behjati, S., Biankin, A.V., Bignell, G.R., Bolli, N., Borg, A., Borresen-Dale, A.L. *et al.* (2013) Signatures of mutational processes in human cancer. *Nature*, **500**, 415-421.

29. Ellrott, K., Bailey, M.H., Saksena, G., Covington, K.R., Kandoth, C., Stewart, C., Hess, J., Ma, S., Chiotti, K.E., McLellan, M. *et al.* (2018) Scalable Open Science Approach for Mutation Calling of Tumor Exomes Using Multiple Genomic Pipelines. *Cell Syst*, **6**, 271-281.

30. Ghandi, M., Huang, F.W., Jane-Valbuena, J., Kryukov, G.V., Lo, C.C., McDonald, E.R., Barretina, J., Gelfand, E.T., Bielski, C.M., Li, H. *et al.* (2019) Next-generation characterization of the Cancer Cell Line Encyclopedia. *Nature*, **569**, 503-508.

31. Landrum, M.J., Chitipiralla, S., Brown, G.R., Chen, C., Gu, B.S., Hart, J., Hoffman, D., Jang, W., Kaur, K., Liu, C.L. *et al.* (2020) ClinVar: improvements to accessing data. *Nucleic Acids Res*, **48**, D835-D844.

32. Tate, J.G., Bamford, S., Jubb, H.C., Sondka, Z., Beare, D.M., Bindal, N., Boutselakis, H., Cole, C.G., Creatore, C., Dawson, E. *et al.* (2019) COSMIC: the Catalogue Of Somatic Mutations In Cancer. *Nucleic Acids Res*, **47**, D941-D947.

33. Turner, T.N., Yi, Q., Krumm, N., Huddleston, J., Hoekzema, K., HA, F.S., Doebley, A.L., Bernier, R.A., Nickerson, D.A. and Eichler, E.E. (2017) denovo-db: a compendium of human de novo variants. *Nucleic Acids Res*, **45**, D804-D811.

34. Li, M.J., Liu, Z., Wang, P., Wong, M.P., Nelson, M.R., Kocher, J.P., Yeager, M., Sham, P.C., Chanock, S.J., Xia, Z. *et al.* (2016) GWASdb v2: an update database for human genetic variants identified by genome-wide association studies. *Nucleic Acids Res*, **44**, D869-876.

35. Clima, R., Preste, R., Calabrese, C., Diroma, M.A., Santorsola, M., Scioscia, G., Simone, D., Shen, L., Gasparre, G. and Attimonelli, M. (2017) HmtDB 2016: data update, a better performing query system and human mitochondrial DNA haplogroup predictor. *Nucleic Acids Res*, **45**, D698-D706.

Supplementary Table 1. Overview of data curated in POSTAR3

Module	Category	Human	Mouse	Zebrafish	Fly	Worm	Arabidopsis	Yeast
CLIPdb	RBP genes (dataset number)	219 (925)	45 (351)	3 (13)	6 (52)	5 (18)	5 (26)	65 (114)
	Sequence motifs	1,530	306	18	36	30	21	378
	Structural preferences	1,481	299	18	36	30	20	364
	Gene Ontologies	110,051	41,685	39	2,998	2,145	1,288	26,042
RBP Binding Sites	All CLIP-seq peaks*	6,320,229	1,482,279	83	108,142	48,214	31,848	326,903
	HITS-CLIP peaks**	6,586,443	113,915	NA	2,122	46	582	5815
	PAR-CLIP peaks**	16,147,424	229,608	NA	420,245	29,940	NA	4,576,445
	iCLIP peaks**	10,349,722	1,288,645	NA	98,267	497,658	188,581	NA
	eCLIP peaks (ENCODE)	2,560,174	NA	NA	NA	NA	NA	NA
	eCLIP peaks (non-ENCODE)**	55,722	NA	NA	NA	NA	NA	NA
	iCLAP peaks**	96	NA	NA	NA	NA	NA	NA
	4SU-iCLIP peaks**	3,290	NA	NA	NA	NA	NA	NA
	urea-iCLIP peaks**	12,464	NA	NA	NA	NA	NA	NA
	BrdU-CLIP peaks**	NA	134	NA	NA	NA	NA	NA
	Fr-iCLIP peaks**	NA	51,267	NA	NA	NA	NA	NA
	circRNA binding sites	4,892,878	NA	NA	NA	NA	NA	NA
	Target gene expression level	12 cell/tissue types	10 cell/tissue types	18 develop-mental stages	30 develop-mental stages	35 develop-mental stages	4 cell/tissue types	3 conditions
RNA Crosstalk	miRNA-binding sites from experiments	3,906,955	1,588,861	NA	NA	NA	NA	NA
	miRNA-binding sites from predictions	12,196,959	7,563,080	NA	1,099,046	671,012	2,524	NA
	RNA modification sites	489,629	495,232	42,463	6,819	NA	20,331	71,466
	RNA editing sites	7,734,919	389,720	6,715	5,037	111,134	NA	NA
Genomic Variants	SNVs	660,146,174	81,432,271	17,153,205	5,618,672	435	486,302	17,153,205
	1000 Genomes SNPs	78,034,351	NA	NA	NA	NA	NA	NA
	Genome Aggregation SNPs	679,192,687	NA	NA	NA	NA	NA	NA
	eQTL SNPs	39,930,484	NA	NA	NA	NA	NA	NA
	sQTL SNPs	16,197,515	NA	NA	NA	NA	NA	NA
Disease Mutations	Cancer COSMIC SNVs	2,371,204	NA	NA	NA	NA	NA	NA
	PanTCGA Whole Exome SNVs	3,572,953	NA	NA	NA	NA	NA	NA
	PCAWG SNVs	23,071,889	NA	NA	NA	NA	NA	NA
	CCLE SNVs	905,616	NA	NA	NA	NA	NA	NA
	Clinically important SNPs	615,108	NA	NA	NA	NA	NA	NA
	GWAS SNPs	127,245	NA	NA	NA	NA	NA	NA
	de novo variants	405,988	NA	NA	NA	NA	NA	NA
	HmtDB variants	6,771	NA	NA	NA	NA	NA	NA
Structurome	Condition	16 cell types / conditions	3 cell types / conditions	NA	NA	NA	1 tissue type	6 cell types / conditions
	PARS records	40,830	NA	NA	NA	NA	NA	5,357
	DMS-seq records	55,062	NA	NA	NA	NA	13,821	10,714
	CIRS-seq records	NA	17,753	NA	NA	NA	NA	NA
	ChemModSeq records	NA	NA	NA	NA	NA	NA	5,357
	DMS-MaPseq records	NA	NA	NA	NA	NA	NA	10,714
	icSHAPE records	26,999	110,800	NA	NA	NA	NA	NA
Translatome	Condition	30 cell / tissue types	11 cell / tissue types	NA	20 stages / cell types	16 cell types / conditions	15 conditions / tissue types	9 conditions
	ORF	3,342,149	2,197,459	NA	1,114,865	914,783	939,162	126,620
Degradome	GMUCT profile	NA	NA	NA	NA	NA	104	NA
	PARE profile	300	14,721	NA	111	NA	34,854	NA

*CLIP-seq peaks analyzed by Piranha

**CLIP-seq peaks analyzed by technology-specific tools

Supplementary Table 2. CLIP-seq datasets added in POSTAR3

(See in excel file)

Supplementary Table 3. ENCODE eCLIP data sets in POSTAR3

(See in excel file)

Supplementary Table 4. Technology-specific and general-purpose peak callers for each CLIP-seq method

CLIP-seq method	Technology-specific peak caller	General-purpose peak caller
HITS-CLIP	CLIPper/CTK*	
PAR-CLIP	MiClip	
iCLIP	PureCLIP	
iCLAP	PureCLIP	
eCLIP	PureCLIP	Piranha
4SU-iCLIP	PureCLIP	
urea-iCLIP	PureCLIP	
BrdU CLIP	CTK	
Fr-iCLIP	PureCLIP	

*CLIPper was used for human HITS-CLIP analysis, while CTK was used for other species.

Supplementary Table 5. structure-seq data sets added in POSTAR3

(See in excel file)

Supplementary Table 6. ENCODE icSHAPE data sets in POSTAR3

(See in excel file)

Supplementary Table 7. Ribo-seq data sets added in POSTAR3

(See in excel file)

Supplementary Table 8. RNA-seq data sets matched with Ribo-seq in POSTAR3

(See in excel file)

Supplementary Table 9. Degradome-seq data sets in POSTAR3

(See in excel file)

Supplementary Table 10. sRNA data sets matched with degradome-seq in POSTAR3

(See in excel file)
